# NDRG1 is induced by antigen-receptor signaling but dispensable for B and T cell self-tolerance

**DOI:** 10.1038/s42003-022-04118-w

**Published:** 2022-11-10

**Authors:** Rose Hodgson, Xijin Xu, Consuelo Anzilotti, Mukta Deobagkar-Lele, Tanya L. Crockford, Jessica D. Kepple, Eleanor Cawthorne, Aneesha Bhandari, Alberto Cebrian-Serrano, Martin J. Wilcock, Benjamin Davies, Richard J. Cornall, Katherine R. Bull

**Affiliations:** 1 MRC Human Immunology Unit, Nuffield Department of Medicine, University of Oxford, Oxford, UK; 2 Wellcome Centre for Human Genetics, Nuffield Department of Medicine, University of Oxford, Oxford, UK; 3 Radcliffe Department of Medicine, University of Oxford, Oxford, UK

**Keywords:** Cancer genomics, Immune tolerance

## Abstract

Peripheral tolerance prevents the initiation of damaging immune responses by autoreactive lymphocytes. While tolerogenic mechanisms are tightly regulated by antigen-dependent and independent signals, downstream pathways are incompletely understood. N-myc downstream-regulated gene 1 (NDRG1), an anti-cancer therapeutic target, has previously been implicated as a CD4^+^ T cell clonal anergy factor. By RNA-sequencing, we identified *Ndrg1* as the third most upregulated gene in anergic, compared to naïve follicular, B cells. *Ndrg1* is upregulated by B cell receptor activation (signal one) and suppressed by co-stimulation (signal two), suggesting that NDRG1 may be important in B cell tolerance. However, though *Ndrg1*^*−/−*^ mice have a neurological defect mimicking NDRG1-associated Charcot-Marie-Tooth (CMT4d) disease, primary and secondary immune responses were normal. We find that B cell tolerance is maintained, and NDRG1 does not play a role in downstream responses during re-stimulation of in vivo antigen-experienced CD4^+^ T cells, demonstrating that NDGR1 is functionally redundant for lymphocyte anergy.

## Abstract (web summary)

Despite an upregulation in anergic B cells, N-myc downstream-regulated gene 1 (NDRG1) is not required for the tolerogenic downstream responses, reducing risk of immune modulation on targeting of NDRG1 for cancer therapeutics.

## Introduction

Balancing the sensitivity required for an effective adaptive immune response, while limiting reactivity to prevent autoimmunity, depends upon an increasingly well-defined series of checkpoints. Lymphocyte anergy is a state of cell-intrinsic functional inactivation, classically described when B or T cells become unresponsive upon antigen-receptor binding by self-antigen^1,2^. This is thought to occur during development when cells are exposed to self-antigen, at insufficient affinity or avidity to induce receptor editing or deletion, or in mature T and B cells when exposed to antigens in the absence of accessory signals in the periphery^3,4^. Central to the fate decision between induction of a tolerogenic or immunogenic response in the periphery is Bretscher and Cohn’s two signal model of cell activation^5^, in which full activation of B and T lymphocytes requires two distinct signals: antigen-engagement of the B cell receptor (BCR) or T cell receptor (TCR) respectively (signal one), which leads to limited initiation of intracellular signaling cascades, and co-stimulation by activated antigen-presenting cell (APC) ligands or CD4^+^ T cell help (signal two), which triggers the sustained, complete signaling required for productive activation^6,7^. If signal two is not received within a temporally-defined window of signal one, this leads to induction of an unresponsive state and cell death, limiting inappropriate responses^3,7^.

In the context of B cell tolerance, immature B lymphocytes that are not eliminated upon encountering low avidity forms of self-antigen in the bone marrow (BM) can undergo phenotypic and biochemical changes to produce a tolerized, functionally-inactive state^3,8,9^. As these anergic B lymphocytes emerge into the periphery, they have an increased threshold for activation, due to proximal blockade of BCR-signaling and selective inhibition of IgM trafficking to the cell surface, classically demonstrated in BCR Ig transgenic (tg) mouse models, including the anti-hen-egg lysozyme (HEL)-specific (Ig^HEL^) model^9,10^. Continual low avidity BCR-signaling in the absence of co-stimulation induces chronic low oscillations of calcium, resulting in sustained sub-optimal activation of ERK and NFAT dependent pathways^11–13^, with proximal blockade of BCR-dependent signaling and a specific block in JNK, Card11, NF-κB^13–15^ and pathways downstream of toll-like receptors (TLRs)^16^. The resultant negative regulatory program diminishes both proliferative responses and capacity for cytokine production in response to stimulation. Furthermore, anergic B cells have a shortened lifespan in a mixed repertoire, due to enhanced dependence on the cytokine B cell activating factor (BAFF) and increased expression of proapoptotic Bim^17–19^. However, through high avidity stimulation of the BCR and in the presence of antigen-specific T cell help, these anergic B cells can initiate differentiation and proliferation and can be recruited into the immune response to foreign antigen^20,21^.

Compared to B cell tolerance, central T cell tolerance by thymic clonal deletion or anergy induction is more robust; however not all self-antigens are presented in the thymus and thus peripheral tolerance plays an important role in preventing autoimmunity^4^. In vivo peptide-induced CD4^+^ T cell anergy has been described as a tolerogenic, autonomous mechanism induced in peripheral T cells that have experienced antigen stimulation in the absence of co-stimulation^4,22^. Characterized by the inability to proliferate or produce cytokines upon re-stimulation, this hypo-responsive state cannot be reversed by IL-2, in contrast to in vitro antigen-experienced T cells re-stimulated with signal one alone^23,24^. Alongside the shared phenotype of clonally anergic CD4^+^ T cells and tolerized B cells, the signaling pathways that characterize the two phenomena also have parallels. In both cell types, the NFAT pathway is selectively activated, modulating negative regulators that induce further downstream anergy factors necessary for the hypo-responsive state^11,12,25,26^. Elucidating the shared and specific molecular mechanisms that block BCR and TCR signaling during the induction of lymphocyte tolerance may unlock new treatments for autoimmune and immunodeficient diseases.

NDRG1 was previously identified as a clonal anergy factor upregulated in naïve and anergic CD4^+^ T cells by signal one alone via the TCR-dependent early growth response genes, *Egr2 and Egr3*, and degraded in a proteasome-dependent manner by co-stimulation. While in vitro induction of NDRG1 expression led to a hypo-responsive state in T cells, the loss of NDRG1 in vivo was described as partially rescuing T cells from peptide-induced tolerance^27^. NDRG1 is a stress- and hypoxia-inducible 43 kDa protein regulating cell growth and differentiation^28,29^, and reported to possess potent anti-metastatic activity in a cell type and tissue-specific manner^30,31^. Though ubiquitously expressed in human tissues, the amount and subcellular localization of NDRG1 expression varies by cell type, with relatively higher staining in kidney, prostate, ovary, intestines and brain. Particular localization to the cytoplasm of Schwann cells in peripheral nerve tissue^32^ is important, since loss of NDRG1 causes sensorimotor neuropathy^33,34^. NDRG1 expression is suppressed via metabolic regulators including N-myc, and the locus is hypermethylated in many cancer tissues including gastric, colon, pancreatic, prostate and some breast cancers^28,35–38^. NDRG1-mediated inhibition of tumor growth and metastasis may explain the anti-cancer effects of iron chelating agents such as Dp44mT^31,39,40^. However positive correlation between tumor tissue NDRG1 levels and cancer progression in some human studies, suggests pleiotropic and context specific roles^41–44^, with silencing of NDRG1 inhibiting migration, invasion and viability of hepatocellular cancer and sarcoma cell lines^45,46^. The potential for pharmacological targeting of NDRG1 in cancer highlights the importance of understanding the role of NDRG1 within adaptive immunity.

In this study, we show that *Ndrg1* is the third most differentially expressed gene in anergic B cells compared to naïve B cells and that the transcriptional regulation of *Ndrg1* parallels that described in stimulated CD4^+^ T cells^27^. Despite this expression pattern, our experiments using a *Ndrg1*^*−/−*^ mouse model demonstrate that NDRG1 is not required for the tolerogenic responses downstream of self-antigen engagement in B cells. In contrast to the observations of Oh et al. when we replicate the experimental protocol of in vivo peptide-induced T cell anergy, we find NDRG1 does not affect immune response or IL-2 production in antigen-experienced CD4^+^ T cells. These findings re-position NDRG1 as a bystander in anergy and have implications for the development of NDRG1 as a therapeutic target in cancer.

## Results

### *Ndrg1* expression is associated with B cell anergy

**Fig. 1:**
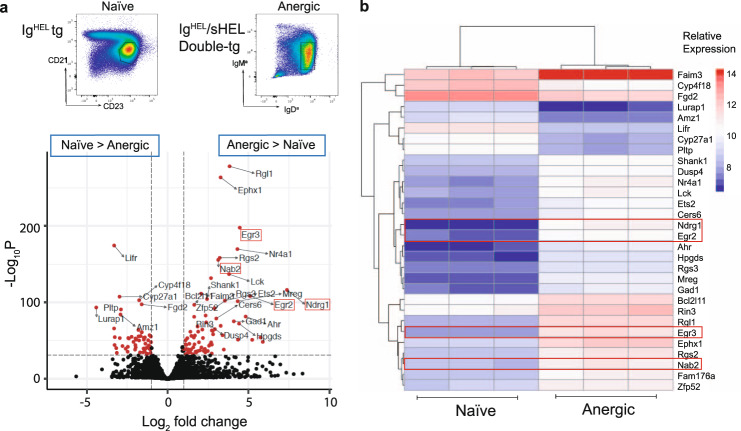
*Ndrg1* is associated with B cell anergy. **a** Top panel: differential gene expression between Ig^HEL^ naïve follicular (B220^+^CD19^+^IgMa^+^CD93^*−*^CD21^mid^CD23^hi^) and Ig^HEL^/sHEL anergic (B220^+^IgMa^lo^IgDa^+^) splenic B cell populations, represented in FACS plots. Lower Panel: Genes differentially expressed >1 and <−1 log_2_ fold change (FC) and below a significance threshold of adjusted *p* value, 10e−32, are shown in red, with the top 30 differentially expressed genes filtered by adjusted *p* value labeled. **b** Heatmap of the relative abundance of the top 30 differentially expressed genes by adjusted *p* value between naïve (left) and anergic (right) B cells. Columns represent samples from individual mice.

To investigate the pathways underlying B cell anergy, we performed RNA Seq on B220^+^CD19^+^IgM^a+^CD93^−^CD21^mid^CD23^hi^ naïve and B220^+^IgM^lo^IgD^a+^ anergic splenic B cell subsets sorted respectively from Ig^HEL^ tg, and double tg mice expressing Ig^HEL^ with or without sHEL as a self-antigen (Ig^HEL^/sHEL) (Fig. 1a). Differential gene expression analysis revealed significant upregulation of anergy-associated transcription factors *Egr2* and *Egr3*, as well as Egr2 dependent targets *Nab2* and *Nrgn*, in anergic compared to naïve B cells, a pattern of expression consistent with NFAT signaling in anergic cells (Fig. 1a, b, Supplementary Table 1)^47^. Of the 868 significantly differentially expressed transcripts, *Ndrg1* was identified as the third most upregulated in anergic B cells by fold change (log_2_ 7.378), and eleventh highest by *p* value (7.41 × 10^−117^ adjusted; Supplementary Table 1). NDRG1 has been described as a CD4^+^ T cell anergy factor downstream of *Egr2/3*^27^, which together with these findings suggested it might be a common participant in the regulation of lymphocyte tolerance. Significant differential expression of *Ndrg1* was verified by qPCR of mRNA extracted from naïve Ig^HEL^ or anergic Ig^HEL^/sHEL B220^+^ cells (1.424 ± 0.1807 vs. 20.05 ± 2.069, *p* value 0.0009), confirming the association between NDRG1 and B cell anergy.

### *Ndrg1* is induced by BCR signaling but suppressed by positive co-stimulation

**Fig. 2:**
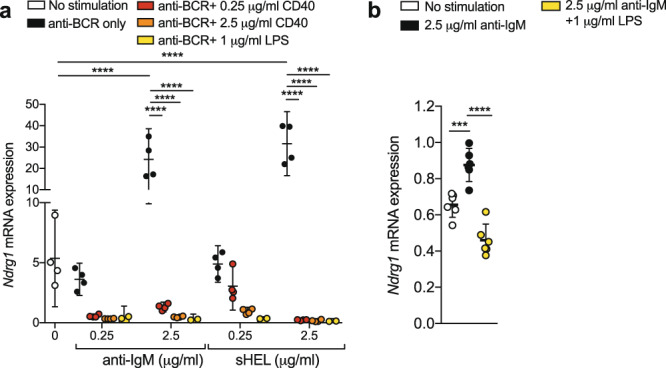
*Ndrg1* expression is regulated by the signal one/signal two axis in B cells. **a** *Ndrg1* mRNA expression calculated relative to *Gapdh* expression in Ig^HEL^ primary B lymphocytes stimulated ex vivo with the indicated concentrations of anti-IgM or sHEL, with or without anti-CD40 or LPS for 24 h at 37 °C. **b** *Ndrg1* mRNA expression calculated relative to *Gapdh* expression in murine A20 Ig^HEL^ tg B cells cultured for 24 h at 37 °C with anti-IgM and with or without LPS. Lines show means with 95% confidence interval (CI) error bars. Data presented are from two to four replicate qPCR readings for each condition and is representative of 3 independent experiments using Ig^HEL^ or non-tg (**a**) or pooled from two A20 (**b**) independent experiments. Significance was determined using a two-way ANOVA with post-hoc Tukey’s testing, ***p < 0.001 and ****p < 0.0001.

In CD4^+^ T lymphocytes, NDRG1 has been proposed as an anergy factor, induced by signal one and inhibited by signal two. To investigate whether *Ndrg1* shared a similar pattern of molecular regulation in B cells, qPCR was performed on mRNA isolated from primary Ig^HEL^ B cells stimulated for 24 h ex vivo via the BCR by anti-IgM or the cognate antigen sHEL (signal one), in the presence or absence of co-stimulation by anti-CD40 or lipopolysaccharide (LPS; signal two). Consistent with the transcriptional control pattern of other anergy factors^8,48^, in vitro antigen-receptor signaling via signal one alone induced *Ndrg1* mRNA expression in primary B cells, but addition of signal two stimulation was associated with a dose-dependent suppression of *Ndrg1* mRNA (Fig. 2a). This pattern of *Ndrg1* expression was also observed in vitro using A20 immortalized B cells engineered to express the Ig^HEL^ BCR (Fig. 2b), supporting the possibility that NDRG1 may play a role in the induction or maintenance of B cell anergy in central or peripheral tolerance.

### A mouse model of NDRG1 deficiency

To explore the role of NDRG1 in B cell immune function, we created a *Ndrg1*^*−/−*^ mouse model using CRISPR/Cas9 directed mutagenesis (Supplementary Fig. 1a). The gene targeting produced an 11 bp deletion within exon 4 of the *Ndrg1* gene in oocytes causing a frameshift mutation *Ndrg1 null* allele, which was propagated further to produce homozygote *Ndrg1*^*−/−*^ mice at expected ratios from heterozygous parents (Supplementary Fig. 1b). PCR-amplification and subsequent Sanger sequencing of cDNA reverse transcribed from RNA isolated from WT and *Ndrg1*^*−/−*^ kidney and splenic B cell lysates demonstrated that the edited RNA transcript is expressed in both tissues (Supplementary Fig. 1c, d) with the 11 bp deletion detectable within the transcript amplified from *Ndrg1*^*−/−*^ kidney and B cell samples with no exon skipping or repair (Supplementary Fig. 1d). NDRG1 protein is expressed at very low levels in WT splenocyte samples and thus undetectable by transitional western blot, however knockout was confirmed by immunoblot analysis of WT and *Ndrg1*^*−/−*^ in kidney lysates using a N-terminal binding antibody (Supplementary Fig. 1e). In splenic lysates, immunoprecipitation and blotting confirmed the complete loss of NDRG1 protein expression, likely a result of nonsense-mediated decay of the altered transcript (Supplementary Fig. 1f).

In humans, *nonsense Ndrg1* mutations are one of the causes of hereditary motor and sensory peripheral neuropathy-type Lom, also known as Charcot-Marie-Tooth disease type 4d (CMT4d)^33^. Consistent with the human disease, and previously reported neurological phenotyping of *Ndrg1*^*−/−*^ mice^34^, our *Ndrg1*^*−/−*^ mice display a shaking, tremorous phenotype with muscle weakness and abnormal clasping of the hind limbs, and reduced proportion of unsupported versus supported rearing (Supplementary Fig. 1g), indicating hind limb weakness and providing further evidence that the protein had been correctly targeted. *Ndrg1*^*−/−*^ mice were smaller than their WT littermate controls, as previously described (Supplementary Fig. 1h)^49^.

### Normal development of the pre-immune repertoire in the absence of NDRG1

**Fig. 3:**
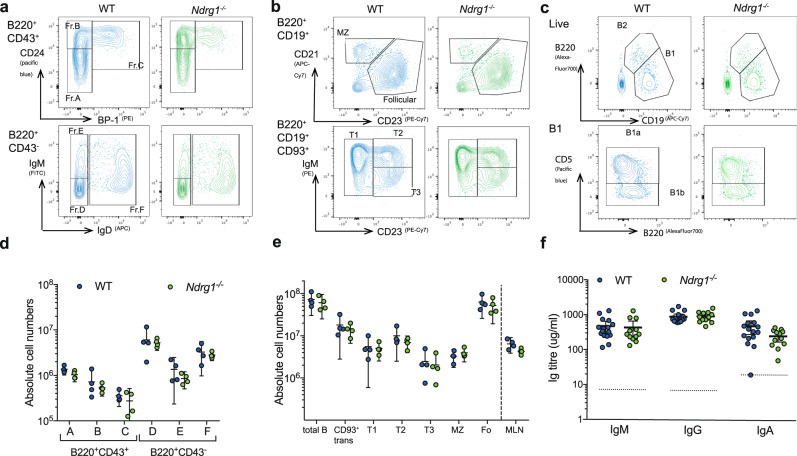
Normal B cell development in the absence of NDRG1. **a** Representative flow cytometry plots of adult WT (blue) and *Ndrg1*^*−/−*^ (green) mice, showing Hardy B cell fractions in BM (**a**), and splenic (**b**) and peritoneal (**c**) B cell subsets. **d** Quantification of absolute BM B cell numbers as gated in (**a**). **e** Quantification of B cell subsets, transitional (trans), transitional populations (T1–T3), marginal zone (MZ) and follicular (Fo) numbers in the spleen and total B cells in MLNs as gated in (**b**). Points are individual mice, lines represent means with 95% CI error bars and are representative of three independent experiments, each with 3–6 mice per group. **f** Serum Ig levels from WT and *Ndrg1*^*−/−*^ mice determined by ELISA. Points are individual mice and lines represent means with 95% CI error bars with 12–16 mice per group. Dotted line represents 1.5× interpolated background absorbance.

Immunophenotyping of *Ndrg1*^*−/−*^ non-tg mice by flow cytometry demonstrated normal development of B cells in the BM (Fig. 3a, d), and no difference in the development, distribution or absolute numbers of peripheral B cell populations within the spleen or absolute B220^+^ B cell numbers in the mesenteric lymph nodes (MLNs) (Fig. 3b, e). Peritoneal B1 B cell populations were also comparable (Fig. 3c), indicating that *Ndrg1* deletion has no effect on development or distribution of unperturbed B cell populations; and serum IgM, IgG and IgA titers were equivalent in *Ndrg1*^*−/−*^ and WT mice (Fig. 3f).

To investigate the requirement for NDRG1 during the response to stimulation by foreign antigens, *Ndrg1*^*−/−*^ and WT B cells were cultured under a range of stimulatory conditions for 24 h and 72 h. The absence of NDRG1 did not alter the in vitro upregulation of activation markers CD69, CD86 or CD25, the extent of proliferation or class-switching in response to TLR ligands (Supplementary Fig. 2a–d).

### Central tolerance and anergy induction during development do not require NDRG1

To explore whether loss of NDRG1 is sufficient to break tolerance to self-antigen at resting state, we tested adult WT and *Ndrg1*^*−/−*^ mouse serum for autoantibodies to nuclear epitopes (ANAs), a sensitive hallmark of autoimmune disease in both mice and humans^50^. There was no increase in ANAs in the absence of NDRG1 expression (WT 1/9, 11.1% positive vs. *Ndrg1*^*−/−*^ 1/9, 11.1% positive, *P* = 1, two-tailed Fisher’s exact test).

**Fig. 4:**
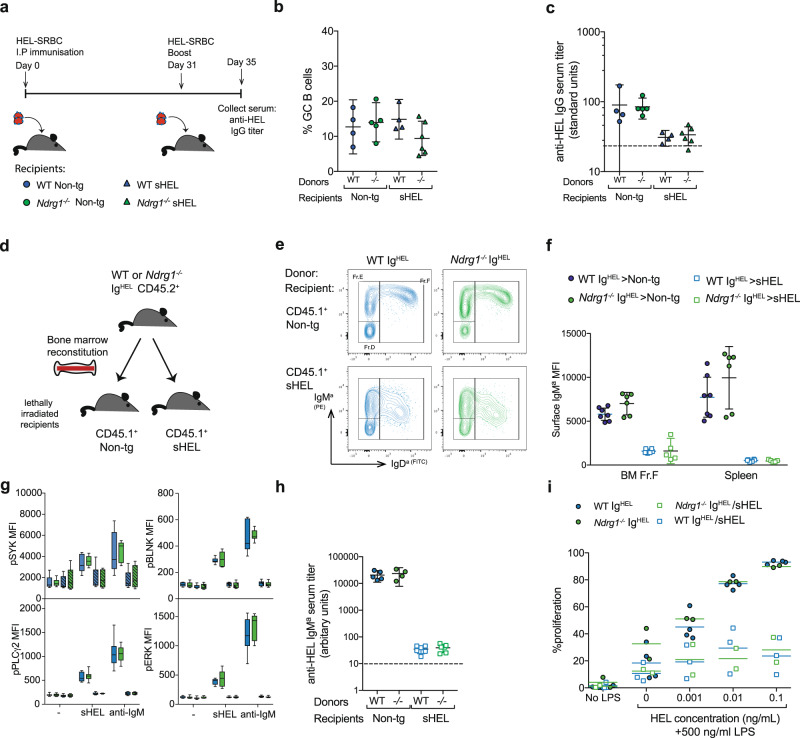
Intrinsic B cell tolerance is intact in the absence of NDRG1. **a** Experimental schematic: WT and *Ndrg1*^*−/−*^ non-tg or sHEL-tg recipients were immunized with HEL-SRBC at day 0 and then boosted with HEL-SRBC again at day 31 before serum was collected on day 35. **b** GC formation on day 35 from mice in (**a**) as determined by gating on GL7^+^CD95^+^ of B220^+^CD19^+^ live lymphocytes. **c** Serum anti-HEL IgG titers from mice in (b) on day 35. Data for (**b**, **c**) are from one experiment with 4–6 mice per group, each point is one mouse and lines are at the mean with 95% CI error bars. Dotted line represents unimmunized background titer level. **d** Experimental schematic: lethally irradiated sHEL or non-tg CD45.1^+^ recipients were reconstituted with WT or *Ndrg1*^*−/−*^ CD45.2^+^ Ig^HEL^ tg BM for 8 weeks. **e** Representative flow cytometry plots of B220^+^CD43^*−*^ BM cells from mice in (**d**). **f** IgM^a^ surface expression on mature recirculating B cells in the BM (B220^+^CD43^*−*^IgD^+^), and on splenic B220^+^CD19^+^ B cells from mice in (**d**), quantified by flow cytometry as MFI. Results are representative of three independent experiments (2–4 mice per group), each point represents individual mice and lines show means with 95% CI error bars. **g** MFIs of phospho-flow intracellular staining of pSyk, pPLCy2, pBLNK and pERK in splenic B220^+^CD19^+^ Ig^HEL^ B cells from WT and *Ndrg1*^*−/−*^ mice in (**d**), after stimulation ex vivo with media alone, 1 μg/ml sHEL or 10 μg/ml IgM. Results are pooled from 3 independent experiments with 2–4 mice per group. Box plots show boxes representing 25th to 75th percentile with a line at median and bars showing min to max. **h** Serum levels of anti-HEL IgM^a^ in (**d**). Each point represents individual mice and lines show means with 95% CI error bars and are representative of two independent experiments. Dotted line shows 1.5× interpolated background. **i** Ex vivo proliferation of B220^+^ splenocytes quantified by CTV dilution in response to media alone, or 500 ng/ml LPS plus sHEL titration for 72 h at 37 °C. Results are pooled from 2 to 3 independent experiments per group, each point represents one mouse.

To isolate and test for a specific defect in B cell tolerance, we investigated whether the provision of T cell help might be sufficient to induce autoantibodies to endogenous self-antigen in the absence of NDRG1. Accordingly, WT or *Ndrg1*^*−/−*^ non-tg and WT or *Ndrg1*^*−/−*^ sHEL mice were primed with HEL-conjugated to sheep red blood cells (HEL-SRBC) and then boosted with HEL-SRBC at 30 days (Fig. 4a). There was no difference in the humoral immune response between WT or *Ndrg1*^*−/−*^ mice in these experiments. As expected^9^, the sHEL expressing mice generated lower HEL-specific IgG titers than non-tg mice, despite equivalent GC formation across groups (Fig. 4b), indicating B cell tolerance to self-antigen, despite the availability of T cell help, remained intact in the absence of NDRG1 (Fig. 4c).

To investigate whether NDRG1 might play a role in tolerance and anergy induction during B cell development in the context of self-antigen, we took further advantage of the Ig^HEL^ and sHEL-tg models. Lethally irradiated CD45.1^+^ non-tg and sHEL mice were reconstituted with WT or *Ndrg1*^*−/−*^ CD45.2^+^ Ig^HEL^ BM (Fig. 4d). Irrespective of NDRG1, flow cytometric analysis showed downmodulation of surface IgM^a^ on mature recirculating B cells in the BM and spleens of sHEL recipients, typical of anergy (Fig. 4e, f); and ex vivo stimulation of the same cells with sHEL showed a block in BCR signaling (Fig. 4g). Anti-HEL-specific IgM^a^ was also equivalently suppressed in sHEL recipients reconstituted with WT or *Ndrg1*^*−/−*^ Ig^HEL^ BM (Fig. 4h). These findings show that NDRG1 is dispensable for the IgM modulation, and block in both proximal BCR signaling and plasma cell differentiation, characteristic of anergic B cells.

Persistent self-antigen stimulation of the BCR in anergic B cells also has the effect of inhibiting proliferative responses to LPS, an essential checkpoint to prevent autoimmunity during exposure to external co-stimulation such as with bacterial infection^16^. To exclude the possibility that NDRG1 might be induced to mediate the inhibition of co-stimulation by LPS, we stimulated CTV-labeled WT and *Ndrg1*^*−/−*^ Ig^HEL^ naïve and Ig^HEL^/sHEL double tg anergic splenic B cells ex vivo with LPS and increasing amounts of sHEL as self-antigen. Proliferation was equivalently inhibited in *Ndrg1*^*−/−*^ anergic B cells compared to WT, indicating that the refractory mechanisms in place to inhibit autoreactive B cell responses to LPS do not depend upon NDRG1 expression (Fig. 4i).

### NDRG1 deficiency does not provide a competitive advantage to anergic B cells within a polyclonal repertoire

**Fig. 5:**
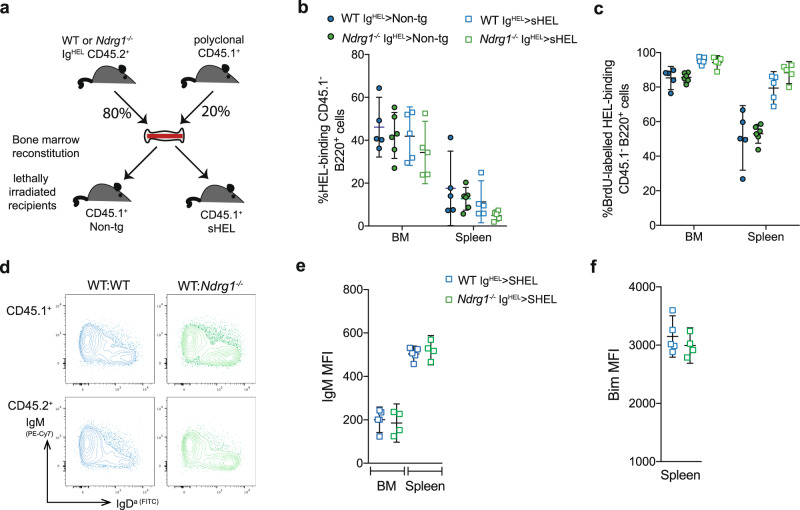
Anergic B cells lacking NDRG1 survive and compete normally in a polyclonal repertoire. **a** Experimental schematic; lethally irradiated WT or sHEL transgenic CD45.1^+^ recipients were reconstituted with 80:20 mixtures of WT or *Ndrg1*^*−/−*^ Ig^HEL^ CD45.2^+^ BM and WT non-tg CD45.1^+^ BM. **b** Percentage of total HEL-binding B220^+^ CD45.1^*−*^ cells in the BM and spleen of mice in (**a**). **c** Percentage of HEL-binding B220^+^CD45.1^*−*^ cells labeled with BrdU after one week. In (**b**, **c**), dots are individual mice from one experiment with 5–6 mice per group, lines show means and 95% CI error bars. d. Representative flow cytometry plots of B220^+^CD43^*−*^ B cells from BM of sHEL mice reconstituted with 50:50 mixtures of WT Ig^HEL^ CD45.1^+^ BM with either WT Ig^HEL^ or *Ndrg1*^*−/−*^ Ig^HEL^ CD45.2^+^ BM. **e** Surface IgM expression on B cell populations gated as in (**d**). **f** Intracellular Bim quantification in B220^+^CD19^+^IgD^a+^splenocytes from (**d**). Each point represents individual mice and lines represent means with 95% CI error bars from one experiment (4–5 per group).

The antigen-induced differential signaling pathways in anergic B cells cause a shortened lifespan in a polyclonal repertoire, due to an inability to compete with non-anergic cells for BAFF, and increased expression of the proapoptotic protein, Bim^17–19^. Since NDRG1 has been implicated in the induction of apoptosis^51^, we hypothesized that NDRG1 might act downstream of signal one to modulate the threshold for survival in a diverse B cell repertoire. To test this, we reconstituted lethally irradiated non-tg or sHEL CD45.1^+^ mice with 80:20 mixtures of *Ndrg1*^*−/−*^ or WT CD45.2^+^ Ig^HEL^ BM and WT non-tg CD45.1^+^ BM (Fig. 5a). Analysis of the reconstituted mice showed equivalent numbers of *Ndrg1*^*−/−*^ and WT Ig^HEL^ B cells in a mixed repertoire (Fig. 5b, Table 1) and an equivalent increase in turnover, in the presence of self-antigen, as judged by BrdU labeling in vivo (Fig. 5c). These data show that NDRG1 is not required to maintain the rapid turnover of anergic B cells that have developed in the presence of self-antigen in the context of competition with non-autoimmune cells for BAFF.

**Table 1:** Anergic B cells lacking NDGR1 survive and compete normally in a polyclonal repertoire.

Tissue	BM (×10^6^)				Spleen (×10^6^)			
Recipient	Non-tg		sHEL		Non-tg		sHEL	
BM donor	WT Ig^HEL^	*Ndrg1*^*−/−*^ Ig^HEL^	WT Ig^HEL^	*Ndrg1*^*−/−*^ Ig^HEL^	WT Ig^HEL^	*Ndrg1*^*−/−*^ Ig^HEL^	WT Ig^HEL^	*Ndrg1*^*−/−*^ Ig^HEL^
Total B	5.86 (1.8)	8.49 (2.5)	8.27 (3.9)	9.17 (1.9)	42.31 (10.9)	71.49 (18.5)	40.95 (14.06)	63.22 (28.1)
Ig^HEL^	2.67 (0.8)	3.45 (1.0)	3.48 (1.7)	3.09 (0.9)	6.56 (3.7)	9.06 (4.3)	4.32 (2.3)	2.82 (1.4)

Numbers of total B220^+^ (Total B) and HyHEL9^+^CD45.1^*−*^ B220+ (Ig^HEL^) cells in the BM and spleen of Non-tg or sHEL recipients reconstituted with 80% WT or *Ndrg1*^*−/−*^ Ig^HEL^ CD45.2^+^ BM and 20% non-transgenic polyclonal WT CD45.1^+^ BM cells. Data reported are means (and SD) from one experiment, with 4–6 mice per group.

Similarly, NDRG1 deficiency did not produce an altered anergic response in B cells exposed to self-antigen in the presence of directly competing WT Ig^HEL^ anergic B cells in a mixed chimera model. Flow cytometric analysis of CD45.1^+^ sHEL recipients reconstituted with equal parts WT CD45.1^+^ Ig^HEL^ BM and either WT Ig^HEL^ or *Ndrg1*^*−/−*^ Ig^HEL^ CD45.2^+^ BM showed comparable levels of surface IgM (Fig. 5d, e), and equivalent Bim expression between *Ndrg1*^*−/−*^ and WT Ig^HEL^ mature B cell populations in the chimeric mice (Fig. 5f).

### NDRG1-deficient B cells exposed to self-antigen in the periphery are subject to follicular exclusion and cell death

Our experiments have demonstrated that NDRG1 is dispensable for the induction and maintenance of anergy when B cells develop in the presence of self-antigen and when challenged with signal two alongside chronic BCR stimulation; however, this does not exclude a role for NDRG1 unique to migration and re-localization of antigen-stimulated B cells, which is linked to their ability to find and interact with T cell help. When naïve B cells first bind antigen, they relocate to the outer periarteriolar lymphoid sheath where they require signal two in the form of T cell help for survival and entry into the follicle to initiate a productive germinal center^19,52^. Similarly, in the context of self-antigen and competing naïve cells, anergic B cells also relocate to the T cell rich cortical zones, where they may be rescued by T cell help; but otherwise typically die from a combination of insufficient engagement with BAFF and increased Bim-dependent apoptosis^17,18,53^.

**Fig. 6:**
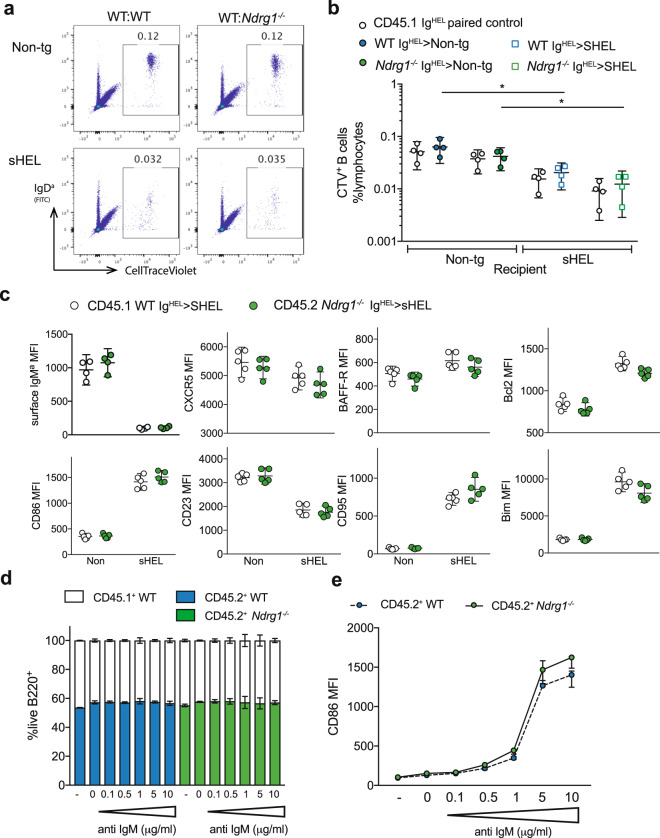
NDRG1-deficient B cells exposed to self-antigen in the periphery are subject to follicular exclusion and cell death. **a** Representative flow cytometry plots of 50:50 mixtures of WT CD45.1^+^ Ig^HEL^ and WT or *Ndrg1*^*−/−*^ CD45.2^+^ Ig^HEL^ splenocytes labeled with CTV dye and transferred by *i.v*. injection into non-tg or sHEL-tg CD45.2^+^ recipients for 48 h, showing live B220^+^CD19^+^ gated populations and % transferred CTV^+^ cells. **b** Percentage of total lymphocytes that were either CD45.1^+^ or CD45.2^+^ CTV^+^ as gated in (a), with white points showing paired CD45.1^+^ Ig^HEL^ cells from donor, colored points are CD45.2^+^. **c** MFI of surface marker expression CD86, CD23, CXCR5, IgM^a^, BAFF-R and CD95, and intracellular Bim and Bcl2, in transferred Ig^HEL^ B cells, gated as in (**a**). Data points are individual mice and lines show means with 95% CI error bars and data are representative from 1 to 3 independent experiments with 4–6 mice per group. **d**, **e** Ratio of B220^+^ cells surviving from 50:50 mixtures of WT CD45.1^+^ and WT or *Ndrg1*^*−/−*^ CD45.2^+^ non-tg B cells stimulated with anti-IgM at the indicated concentrations for 16 h at 37 °C ex vivo (**d**) and CD86 surface expression as MFI on the CD45.2 + B220^+^ cells (**e**). Data are representative of 3 independent experiments with columns or circles as means with 95% CI error bars.

To exclude a possible role for NDRG1 in these interactions, equal ratios of WT Ig^HEL^ CD45.1^+^ and WT or *Ndrg1*^*−/−*^ Ig^HEL^ CD45.2^+^ splenocytes were labeled with CTV dye and transferred into non-tg or sHEL CD45.2^+^ recipients (Fig. 6a). After 48 h, the total number of transferred CTV^+^ HEL-binding Ig^HEL^ B cells were significantly reduced in sHEL compared to non-tg recipients, but ratios of CD45.1^+^:CD45.2^+^ surviving transferred HEL-binding cells were equivalent for WT and *Ndrg1*^*−/−*^ donor populations (Fig. 6b). These findings show that NDRG1 does not play a role in the elimination of signal one experienced B cells in the periphery. This conclusion is supported by the equivalent induction of CD95, BAFF-R, Bcl2 and Bim proteins in the CD45.1^+^ WT and CD45.2^+^ *Ndrg1*^*−/−*^ antigen-experienced B cells (Fig. 6c). In sHEL recipients, IgM^a^ downmodulation and upregulation of the activation marker CD86 were similar in CD45.1^+^ WT and CD45.2^+^ *Ndrg1*^*−/−*^ Ig^HEL^ B cells (Fig. 6c). Levels of localization markers CD23 and CXCR5 were also reduced equivalently on exposure to sHEL in CD45.2^+^ *Ndrg1*^*−/−*^ antigen-experienced B cells, suggesting follicular exclusion is also unlikely to depend on NDRG1 expression (Fig. 6c).

To further confirm that NDRG1 has no role in inducing apoptosis downstream of signal one in the absence of signal two, mixed cultures of WT CD45.1^+^ and WT or *Ndrg1*^*−/−*^ CD45.2^+^ non-tg B cells were stimulated with a titration of plate-bound anti-IgM for 16 h in vitro^18^. The ratio of CD45.1^+^:CD45.2^+^ B cells was not altered by increasing concentrations of anti-IgM in cultures (Fig. 6d), despite equal antigenic engagement as measured by CD86 upregulation (Fig. 6e), supporting our conclusion that NDRG1 does not induce B cell death in response to signal one in the absence of signal two.

### Antigen-experienced T cells responses are not altered in the absence of NDRG1

The finding that NDRG1 is either dispensable or acts redundantly for the induction and maintenance of B cell anergy, led us to re-examine the evidence for NDRG1 regulation of induced T cell anergy. As previously reported^27^, NDRG1 deletion had no effect on thymocyte development (Supplementary Fig. 3a, c) or maturation and distribution of peripheral T populations (Supplementary Fig. 3b, d).

**Fig. 7:**
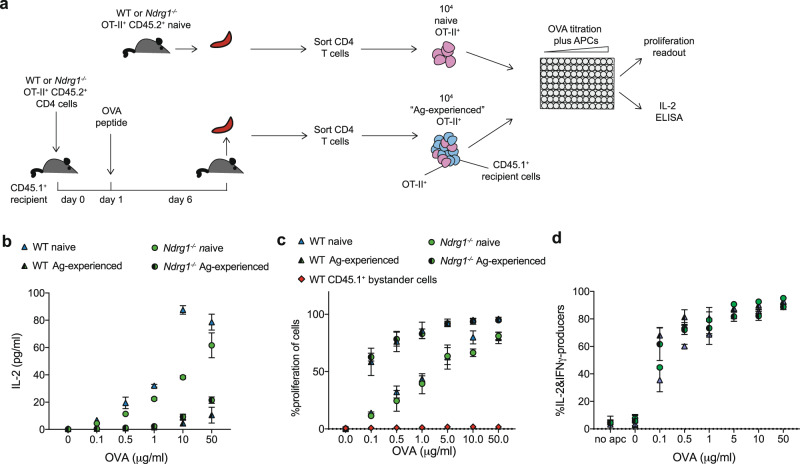
Antigen-experienced T cells respond normally in the absence of NDRG1. **a** Experimental schematic; OVA-specific CD4^+^ OT-II^+^ T cells were isolated from CD45.2^+^ WT or *Ndrg1*^*−/−*^ OT-II^+^ tg mice and adoptively transferred to CD45.1^+^ non-tg recipients, with subsequent intravenous injection of 500μg OVA peptide. After 5 days, splenic CD4^+^ cells were isolated from recipient mice and naïve unstimulated WT or *Ndrg1*^*−/−*^ OT-II^+^ tg mice and loaded with CTV. Equal numbers of Vα2^+^ CD45.2^+^OT-II^+^ cells were cultured in vitro with a titration of OVA peptide for 72 h at 37 °C with irradiated APCs. **b** IL-2 concentration in culture supernatants in (**a**). **c** Proliferation of CD4^+^Vα2^+^ CD45.2^+^OT-II^+^ T cells and non-tg bystander WT CD45.1^+^ cells (red) as measured by CTV dilution by flow cytometry. **d** Quantitation of intracellular IL-2 and IFNγ production by flow cytometry within OT-II^+^ CD4^+^ T cells from (**a**). Points represent means of technical replicates from one (naïve) or two (antigen-experienced) mice with error bars representing standard deviation. Results are representative of two (**b**), three (**c**) or one (**d**) independent experiment(s).

To confirm that NDRG1 acts as a T cell-specific anergy regulator, we recapitulated the previously described in vivo peptide-induced T cell anergy assay^27,54^ using our mouse model of NDRG1 deficiency. In these experiments, CD45.2^+^ ovalbumin (OVA)-specific TCR tg CD4^+^ OT-II^+^ T cells were isolated from WT or *Ndrg1*^*−/−*^ OT-II^+^ tg mice and adoptively transferred into CD45.1^+^ congenic recipients, with subsequent intravenous injection of endotoxin-free OVA peptide on day 1. After 5 days, naïve and challenged splenic WT and *Ndrg1*^*−/−*^ OT-II^+^ CD4^+^ T cells were harvested and stimulated in vitro (Fig. 7a).

As reported previously^27,54^, IL-2 concentration in the culture supernatant from antigen-experienced CD4^+^ T cells was lower compared to that from naïve cells (Fig. 7b; *p* = < 0.05 at all concentrations of OVA). However, we could not replicate the reported increase in IL-2 in *Ndrg1*^*−/−*^ relative to WT antigen-experienced T cells^27^ (Fig. 7b). In contrast to some previous findings using a thymidine incorporation assay, flow cytometric analysis of CTV dilution reproducibly demonstrated a significantly enhanced proliferation response in antigen-experienced OT-II^+^ T cells compared to naïve (Fig. 7c; *p* = < 0.05 between 0.1 and 5 μg/ml OVA), irrespective of NDRG1 expression. Bystander WT CD45.1^+^ non-tg cells demonstrated no proliferation in cultures containing either WT or *Ndrg1*^*−/−*^ OT-II^+^ cells at any concentration of OVA (Fig. 7c, red points).

We therefore considered that the assay may represent a stimulatory response rather than anergy, and hypothesized that the lower IL-2 in the supernatant of antigen-experienced cells may consequently reflect a higher rate of consumption due to greater proliferative responses of these cells compared to cultures containing naïve cells. To test this, production of cytokines IL-2 and IFNγ was directly measured by intracellular staining. Ag-experienced populations produced more IL-2 and IFNγ in a dose-dependent manner than naïve CD4^+^ T cells, consistent with a stimulatory rather than anergic response (Fig. 7d, *p* = < 0.05 at 0.1–0.5 μg/ml OVA), a response that was unaffected by NDRG1 deficiency (Fig. 7d).

## Discussion

The development of more effective and better tolerated treatments for cancers, autoimmune and immunodeficiency diseases relies on defining the key molecular mechanisms that distinguish productive from tolerogenic lymphocyte responses^8^. Despite the success of many monoclonal therapies targeting immune checkpoints, they are associated with an increased risk of autoimmune adverse events^55–57^. Pre-clinical investigation into the immune consequences of anti-cancer therapy is therefore key to preventing serious immunopathology. NDRG1 is a stress- and hypoxia-inducible regulator of cell growth and differentiation^28,29^, implicated in some contexts as a metastasis inhibitor due to involvement within multiple signaling pathways^30,31^. Consistent with this, anti-cancer iron chelators, Dp44mT and DFO may act via NDRG1-upregulation^31,39,40^. Pertinently for this study, in multiple human cancers increased tissue expression of NDRG1 has been associated with poor outcomes^41–44^, with in vitro evidence for suppression of NDRG1 as a novel anti-cancer strategy^45,46^. The therapeutic targeting of NDRG1 presents a promising treatment opportunity, but requires clarification of the immune effects of NDRG1 inhibition.

In 2015, *Ndrg1* was described as a T cell anergy factor, regulated by the signal one/signal two axis, induced downstream of NFAT and *Egr2* upon TCR signaling and repressed by co-stimulation. *Ndrg1*^*−/−*^ CD4^+^ T cells were reported to be partially resistant to peptide-induced anergy in vivo^27^. Selective activation of the calcium-regulated transcription factor NFAT, and *Egr* family driven gene signatures, are known to contribute to both B and T lymphocyte unresponsiveness^11,12,25,26^, with defects in maintenance of T and B cell tolerance, including increased production of self-reactive Ig, in *NFAT1*^*−/−*^ mice^12,58^. Our findings identified *Ndrg1* as the third most upregulated gene in anergic B cells compared to naïve cells. The evidence for shared genetic regulation of *Ndrg1* by the signal one and two costimulatory axis in B and T cells^27^ suggested that NDRG1 could represent a potential central factor regulating lymphocyte tolerance downstream of *Egr2/3* and NFAT activation.

Our experiments employing a CRISPR/Cas9 generated NDRG1-deficient mouse demonstrated that NDRG1 is either dispensable or acts redundantly within tolerance mechanisms during B cell development and activation. NDRG1 deficiency did not affect induction and maintenance of B cell anergy in the Ig^HEL^ > sHEL BM chimeric model, nor did it alter the maintenance of cell-intrinsic tolerance to self-antigen at resting state, upon immunization or during direct competition. We hypothesized that NDRG1 action may be limited to specific properties of B cell tolerance such as influencing the threshold for breakdown of tolerance in response to external stimulation, as *Ndrg1* expression is closely regulated by signal two in B and T lymphocytes; however, the characteristic inhibition of signal two was intact in *Ndrg1*^*−/−*^ anergic B cells. Furthermore, the loss of NDRG1 had no effect on the response of naïve splenic B cells exposed to self-antigen, or the induction of cell death due to the absence of rescue by signal two. There was no requirement for NDRG1 in these aspects of B cell tolerance, despite clear regulation of NDRG1 by the signal one/signal two axis in lymphocytes.

To strengthen the findings from our mouse model of NDRG1 deficiency, and with the aim of demonstrating that NDRG1 functions in CD4^+^ T cell tolerance, we recapitulated an experimental design to show the role of NDRG1 in in vivo peptide-induced T cell anergy^27^. In contrast to the results reported previously, we saw increased proliferation of endotoxin-free peptide antigen-experienced in vivo pre-stimulated CD4^+^ T cells, as would be expected during re-stimulation rather than an anergic response. The reported reduction in IL-2 in culture supernatant upon re-stimulation of antigen-experienced CD4^+^ T cells was reproducible, but NDRG1 deficiency did not affect this phenomenon. On further exploration, we found that the intracellular levels of IL-2 and IFNγ were higher in the antigen-experienced cells, consistent with increased consumption of cytokines associated with a higher proliferative response on re-stimulation, explaining the reduced IL-2 detected in the culture supernatants of these cells.

The in vivo peptide-induced T cell anergy model is well described and characterized by reduced IL-2 in culture supernatant^22,54,59^, though the response to IV peptide may be variable^22^, and is not consistent in non-chimeric OT-II tg mice^54^. Our use of CTV viable proliferation dye in contrast to thymidine incorporation to measure proliferation accounts for any disproportionate loss or inhibition of OT-II^+^ CD4^+^ T cells during in vitro culture. Though we find that non-tg cells from the transfer recipient contribute minimally to proliferation in antigen-experienced conditions, we cannot exclude a suppressive or cytotoxic effect on antigen-experienced OT-II^+^ cells from non-tg peptide experienced cells in vitro in previously described experiments; such effects have been shown to inhibit proliferation in an OT-I^+^ CD8^+^ in vivo peptide model^60^. Together, these experiments suggest that this experimental system may not reliably test for peptide-induced CD4^+^ T cell unresponsiveness and call into question the proposed role of NDRG1 in vivo T cell anergy.

In contrasting our results with the previous observations in T cells, we also note that where the model of NDRG1 deficiency described here was generated using CRISPR/Cas9 on a pure C57BL/6 background, the *Ndrg1*^*−/−*^ mouse used in previous experiments exploring T cell anergy^27^ was created using 129 ES cells^34^, a method which can be associated with co-transfer of immunomodulatory genes alongside the mutant gene^61^. Although the mice were backcrossed for 8 generations^27^, a 129 strain-specific immune effect cannot be fully excluded.

Taken together our results position NDRG1 as a bystander, which may solely represent a biomarker of lymphocytes chronically stimulated by signal one alone. An alternative possibility is that anergy is maintained in the absence of NDRG1 due to functional redundancy. Mammals have 4 NDRG1 proteins, which share 53–65% homology^62,63^ and transcriptional repression via myc. The preservation of central nerves despite peripheral demyelination in CMT4d has been attributed to compensation by NDRG2–4^64^, However, it is not clear if any such compensation is relevant outside the nervous system, and notably NDRG2–4 are not upregulated in anergic B cells (Fig. 1).

When the anergic B cell engages antigen, failure to recruit Syk family kinases to the BCR^65^ results in Lyn mediated inhibition of the PI3K pathway via SHIP-1 and PTEN^66,67^. NDRG1 has been shown to exist in a positive feedback loop with PTEN, whilst inhibiting PI3K^31,68^. However, the lack of redundancy with loss of either SHIP-1 or PTEN suggests that NDRG1 does not act reciprocally with these proteins^66,67^. In addition to a role in PI3K/AKT signaling, evidence from the cancer field indicates that NDRG1 can also modulate the TGFβ/SMAD and RAS/RAF/MEK/ERK axes, inhibit NF-κB and the ErbB receptors, suppress sonic hedgehog and modulate the phosphorylation of Cbl-b^31,69^, Cbl-b itself associated with maintenance of B and T cell tolerance^70,71^. NDRG1 also inactivates canonical Wnt/β-catenin signaling in BM stromal progenitor cells and cancer cells, while promoting β-catenin plasma membrane expression^8,9^. Stable β-catenin expression on CD4 T cells has been linked to an unresponsive anergic like phenotype, while uncontrolled β-catenin accumulation in development induces B cell anergy^10,11^. Furthermore, an independent compensatory role for NDRG1 within lymphocytes in pathways such as PI3K, Src or Wnt/β-catenin cannot be fully discounted; transcriptomic or proteomic profiling of *Ndrg1*^*−/−*^ anergic B cells in comparison to WT anergic, and *Ndrg1*^*−*/*−*^ naïve B cells may identify alterations in such pathways.

Beyond tolerance, there is in vitro and in vivo evidence for immunomodulatory NDRG1 function in mast cells, where it appears to promote degranulation response to stimuli^72,73^. However, while NDRG1 deficiency is a well-described cause of CMT in humans, autoimmunity or immunodeficiency are not described in CMT4d^33,74–76^.

The regulation of NDRG1 points to a central role in the cellular stress response. In addition to N-myc and C-myc, NDRG1 is upregulated after DNA damage via p53^51^, by hypoxia via HIF1α^40^ and *Egr1*^77^, and by PTEN^68^. While the modulators of NDRG1 specific to B cells have not been well characterized, several known drivers of the anergic versus activation axis in B cells are linked to NDRG1 regulation. These include transcription factors, *Egr2* and *Egr3*, downstream of NFAT activation in B and T cell anergy^47,78^, of which *Egr2* has been shown to regulate NDRG1 in T cell clones^27^. A role for HIF1α in both B cell development^79^ and in regulating B cell tolerance^80^ has been reported and hypoxia and HIF1α-regulation of NDRG1 is well characterized^31^. Downstream of BCR and BAFF-R stimulation, via PTEN/PI3K, the NF-κB pathway is key for B cell activation, proliferation and survival; Rictor, an essential mTOR component, is an NDRG1 regulator^81^, and Rictor-deficient B cells have impaired BCR and BAFF-R engagement and associated reduced AKT (273) and NDRG1 phosphorylation^82^. These shared regulatory pathways between B cell anergy and NDRG1 suggest possible explanations for the observed correlation between NDRG1 levels and anergy.

In conclusion, we found no evidence that NDRG1 has any functional role during antigen-receptor signaling in B  or T lymphocyte populations. We have shown that, while NDRG1 is dispensable for B cell tolerance, the association of NDRG1 expression with B cell anergy represents a biomarker for anergic cells, stimulated by the BCR in the absence of signal two, and reminiscent of other cell types under conditions of cell stress^29,31,34,51,83–85^. The fact that NDRG1 expression was not required for the induction or maintenance of B cell anergy, or other processes of chronic BCR stimulation, enhances the potential for targeting NDRG1 for human anti-cancer treatment.

## Methods

### Mice

All procedures involving animals were performed in accordance with the Animals (Scientific Procedures) Act 1986, amended 2012, with procedures reviewed by the clinical medicine Animal Care and Ethical Review Body and conducted under Home Office Project License, P79A4C5BA. Mice were housed in individually ventilated cages, provided with food and water ad libitum and maintained on a 12 h light:12 h dark cycle (150–200 lux). The only reported positives on FELASA health screening over the entire time course of these studies were for *Helicobacter* spp*, Chilomastix Sp, Enteromonas muris, Trichomonas Sp, mouse Norovirus*, and *Entamoeba* spp.

C57BL/6JOlaHsd mice were purchased from Envigo. Ig^HEL^ (C57BL/6-Tg(MD4)4Ccg/J), soluble HEL (sHEL) tg (C57BL/6-Tg(ML5)5Ccg/J) and OT-II tg mice (C57BL/6-Tg(TcraTcrb) 425Cbn/Crl) have been described previously and were maintained on a C57BL/6 background^9,86^. *Ndrg1*^*−/−*^ mice were generated by CRISPR/Cas9 directed mutagenesis on the Ig^HEL^ background as described in Supplementary Fig. 1a in collaboration with Dr Ben Davies, Transgenics Core, Wellcome Center for Human Genetics. CRISPR/Cas9 nuclease guide (5’GATGACAGGACGGTTGCCCTTGG) was designed against exon 4 of the *Ndrg1* gene (ENSMUSG00000005125)^87^. Exon 4 is the most upstream exon of the *Ndrg1* gene for which, if exon skipping occurred despite mutagenesis, the resulting transcript would still lead to nonsense-mediated decay. Complementary oligos for the guide sequence (5′-CACCGATGACAGGACGGTTGCCCT-3′, 5′-AAACAGGGCAACCGTCCTGTCATC-3′) were annealed, creating a compatible linker for cloning into CRISPR/Cas9 vector pX330-Puro, pre-digested with BbsI^88^. A template for in vitro transcription using a T7 RNA polymerase was generated from this plasmid by PCR and used for guide-RNA preparation using the EnGen sgRNA synthesis kit (NEB), followed by purification of the resulting RNA with the Megaclear Clean-up kit (Ambion). Guide-RNA efficiency and specificity was tested by lipofection into murine melanocyte, B16F10 line. C57BL/6J oocytes derived from Ig^HEL^ studs were microinjected with 20 ng/μl guide-RNA and 20 ng/μl Cas9 mRNA before reimplantation into pseudo-pregnant foster mothers at the two-cell stage. Founder mice harboring an indel deletion in *Ndrg1* were identified by PCR and Sanger sequencing using the following primers, *Ndrg1* genomic set 1 for PCR and Sanger Sequencing; forward 5′-GGACTGTGCTTGTATGACATTC-3′, reverse; 5′-GTGTCCATAGTCAGTGGGTCAG-3′, *Ndrg1* genomic set 2 PCR; forward 5′-CCAAACTCACGGTTCATGCC-3′, reverse 5′-CAGGTGATGGGCCTCTGTCT-3′, amplifying a 522 bp region and 151 bp region of *Ndrg1* exon 4, respectively. Founder mice were then backcrossed for 7 generations with wild-type (WT) C57BL6/J mice, and intercrossed to generate homozygotes, selecting both *Ndrg1*^*−/−*^ Ig^HEL^ tg and *Ndrg1*^*−/−*^ non-tg animals for experiments. The following primers sets were used for PCR and Sanger sequencing of Ndrg1 mRNA, mRNA set 1 spanning whole CDS; forward 5′-ATGTCCCGAGAGCTACATG-3′ and reverse 5′-TTAGCAGGACACCTCCATGG-3′ and mRNA set 2; forward 5′-ATGTCCCGAGAGCTACATG-3′ and reverse 5′-AGTTGAAGAGGGGGTTGTAG-3′.

### Sheep red blood cell (SRBC) immunization

HEL-SRBC were prepared by incubating SRBC in Alsever’s solution (FirstLink) with 20 mg HEL per 10 ml 10% SRBC in conjugation buffer (0.35 M Mannitol, 0.01 M NaCl in HBSS) and later addition of 100 mg EDCI (Sigma Aldrich). After washing with HBSS, 200 μl 2 × 10^9^ HEL-SRBC were injected intraperitoneally into each mouse.

### BM chimeras

For BM chimeras, sHEL and non-tg CD45-1^+/+^ or CD45-1^+/*−*^ recipients were irradiated with two doses of 4.5 Gy, spaced by 3 h, and intravenously injected with at least 5 × 10^6^ *Ndrg1*^*−/−*^ Ig^HEL^ or WT Ig^HEL^ BM cells. All chimeras were given water treated with 1% Enrofloxacin antibiotic (Baytril, Bayer) for the first 3 weeks of reconstitution. BM chimeras were allowed to reconstitute for at least 7 weeks before immunization or analysis. Specified mice received BrdU supplemented with 1% sucrose for 7 days in their drinking water at a final concentration of 0.8 mg/ml.

### Hind limb strength assessment

Hind limb strength was measured by observing over 5 min the number of times an animal reared to stand, placing weight onto hind legs, either unsupported or supported, the ratio of unsupported/supported rearing was then calculated.

### Ex vivo culture and stimulation

Total B cells were isolated from RBC-lysed splenocyte suspensions by positive selection using Miltenyi Biotec MACS LS columns and CD45R (B220) microbeads. B220^+^ or B220^*−*^ splenocytes were cultured at a final density of 1–2 × 10^5^ cells/mL in complete R10 (RPMI media with 10% FCS plus non-essential amino acids, 1 mM sodium pyruvate and 50 µM 2-β-mercaptoethanol, 20 mM hepes, 2mM L-glutamine, 10units/mL penicillin, 10 µg/mL streptomycin and 20 µg/ml neomycin) at 37 °C, 5% CO_2_. For RNA extraction, cells were washed with PBS after 24 h culture and pelleted for later RNA extraction as described below. For proliferation analysis, 10^6^–10^7^ B220^+^ or B220^*−*^ cells were labeled with 2.5 µM CellTraceViolet (CTV) dye before culturing for 72–96 h prior to analysis by flow cytometry as below. The following stimuli were prepared as indicated in complete R10: anti-IgM, µ-chain specific (Jackson ImmunoResearch; 115-005-075), LPS from Salmonella species (Sigma Aldrich; L7770), HEL (Sigma Aldrich; L6876-5G), anti-CD40 (Biolegend; 102908), anti-CD3e (Biolegend; 100331), anti-CD28 (Biolegend; 122004) and recombinant mouse IL-4 (Biolegend; 715004).

### A20 Ig^HEL^ tg cell line

A20 is a Balb/c B cell lymphoma line, originally sourced from ATCC, a gift from Professor Simon Davis, University of Oxford. HyHEL10 IgM heavy chain and kappa light chain were cloned from Ig^HEL^ mouse cDNA into pHR-SIN-CSGW vector under the control of the spleen focus forming virus promotor, then transfected into A20 B cells in which the endogenous BCR had been targeted by CRISPR-Cas9 gene editing. HyHEL10 BCR surface expression was confirmed by flow cytometry.

### Flow cytometry

Cell suspensions were isolated in RPMI media containing 2% fetal calf serum (FCS), 10–20 mM Hepes and cells counted using WBC counting fluid (1.5% acetic acid, 0.5% methyl violet in water) and a haemocytometer^89^. If required for downstream analyses, samples were RBC-lysed. 0.5–2 × 10^6^ cells were aliquoted and washed with 200 μl FACS buffer (PBS with 2%FCS, 20 mM Hepes, 0.05% sodium azide). Cells were stained in antibody staining cocktail, then washed for acquisition. HEL-binding cells were detected by pre-incubation with 200 ng/ml HEL, washed with FACS buffer, then counterstained with HyHEL9 conjugated to pacific blue or FITC in house^89^. Data were collected on a BD FACS CANTO10c. The following antibodies used during flow cytometric staining were from Biolegend; anti-B220 (1:400–500, 103236, 103232, 103212, 103243), anti-CD19 (1:400, 115528, 115530), Zombie Aqua live/dead (1:200, 423102, 423106), anti-IgM (1:400–500, 406508, 406512), anti-IgD (1:400, 405704, 405716, 405708), anti-IgD^a^ (1:400, 406104), anti-IgM^a^ (1:600, 408608), anti-CD23 (1:250, 101614), anti-CD93 (1:100, 136510), anti-CD21 (1:400, 123418, 123412), anti-CD24 (1:400, 101820, 101836), anti-CD5 (1:100, 100629), anti-CD86 (1:400, 105028), anti-CD44 (1:100, 103020, 103006), anti-CD25 (1:500:800, 102008), anti-CD4 (1:400, 100430), anti-CD8a (1:400, 100734), anti-CD62L (1:100, 104412), anti-CD3 (1:100, 100214, 100330, 100328), anti-CD69 (1:200, 104530), anti-CD45.1 (1:200, 110730, 110722, 110708), anti-CD45.2 (1:200, 109841, 109818, 109824), anti-BAFF-R (1:200, 134103), anti-CD95 (1:200, 152604) and anti-TCRVα2 (1:200, 127806). The following antibodies were from BD Pharmingen; anti-CD21 (1:400, 563176), anti-BP-1 (1:100, 553735), anti-IgM^a^ (1:500, 553516), anti-CD43 (1:100, 562865), anti-IgM (1:500, 553437), phospho-PLCγ2 (1:25, 558498), phospho-BLNK (1:25, 558443), anti-BrdU (1:20, 364108), anti-CXCR5 (1:200, 145504), anti-Bcl2 (1:200, 633506), anti-Bcl2 quantification kit (1:5, 556537), anti-CD95 (1:200, 557653) and anti-IgG-1 (1:200, 563285). The following antibodies were from eBioscience anti-IgM (48-5890-82), anti-CD4 (1:400, 25-0041-82), phospho-ERK (1:50, 53-9109-42) and phospho-SYK (1:50, 12-9014-42). CellTraceViolet cell proliferation kit (C34557) was from ThermoFisher. Anti-Bim was from CST NEB (1:100, 10408S).

### Phospho-FLOW and intracellular staining

2–5 × 10^6^ splenocytes were stimulated with 1μg/ml sHEL or 10μg/ml anti-IgM F(ab)_2_ (Jackson Immunoresearch) at 37 °C for 5 min and subsequently fixed and permeabilized using the BD Cytofix/Cytoperm kit in combination with Cytofix and Perm Wash buffer (BD Biosciences; 554655, 554722, 557885) before staining in intracellular antibody staining cocktail.

To detect BrdU incorporation, surface marker stained cells were fixed using BD Cytofix/Cytoperm, treated with BD Cytoperm Permeabilization Buffer Plus (BD Biosciences; 561651) by protocol, and fixed again before treatment with 30μg DNase/10^6^ cells for 1 h at 37 °C. Cells were further incubated with fluorescent anti-BrdU prior to acquisition.

### Fluorescent activated cell sorting (FACS)

Splenic B220^+^ CD19^+^ CD21^mid^ CD23^hi^ naïve follicular B cells were isolated from Ig^HEL^ tg mice and splenic B220^+^ IgMa^lo^ IgDa^+^ anergic B cells were sorted from Ig^HEL^/sHEL double tg mice. Cell populations were surface stained and sorted with a FACS sorter (BD-AriaIII), and collected in ice-cold medium (50% FCS).

### RNA-Sequencing (RNA Seq) and Transcriptomic Analysis

RNA data^90^ are deposited in Gene Expression Omnibus under accession number, GSE135650. DESeq2 v.1.28.1 was used for differential expression analysis between follicular and anergic B cell populations^91^. Counts were transformed using variance stabilizing transformation for visualization in DESeq2. A gene was considered differentially expressed if the log2 fold change was > = ±1 and adjusted *p* value <0.05.

### Quantitative PCR (qPCR)

RNA was extracted from homogenized murine kidney lysates or 10^6^–10^7^ pelleted cells using the Qiagen RNeasy Plus kit (Qiagen; 74134) or using TRIzol extraction (Life Technologies; 12183555) by protocol. Equivalent quantities of RNA were reverse transcribed to cDNA using SuperScript II Reverse Transcriptase (ThermoFisher Scientific; 18064014), priming with Oligo(dT12–18) primer (ThermoFisher; 18418012). Expression of *Ndrg1* and *Gapdh* mRNA was quantified by qPCR using SYBR Green PowerUp Master Mix (ThermoFisher Scientific; A25776) with the following primers *Ndrg1* qPCR forward 5′-ACCCTGAGATGGTAGAGGGTCTC-3′, reverse 5′-CCAATTTAGAATTGCATTCCACC-3′, *Gapdh* qPCR forward 5′-TGTGTCCGTCGTGGATCTGA-3′, reverse 5′-TTGCTGTTGAAGTCGCAGGAG-3′, and run on a Bio-Rad CFX96 detection system. Standard curve construction demonstrated equivalent efficiencies of *Ndrg1* and *Gapdh* cDNA amplification, therefore changes to *Ndrg1* mRNA levels were directly calculated relative to endogenous *Gapdh* expression using the following equation: 1/(2^(*Ndrg1* C_t_ – *Gapdh* C_t_))*100.

### Western blot

Kidney protein lysates were produced by homogenization in lysis buffer containing 50 mM Tris-HCl pH7.4, 0.5%NP40, 150 mM NaCl, 20 mM MgCl_2_, protease and phosphatase inhibitors (Sigma Aldrich; 4906837001/5892970001), or in supplemented RIPA buffer (Santa Cruz; sc-24948). 1–25 μg protein lysate was reduced by addition of SDS-containing reducing buffer containing 0.1 M DTT and denatured by boiling at 95 °C for 5 min and loaded onto 4–12% Tris-HCl gels (ThermoFisher; NP0323BOX). Proteins were transferred to a nitrocellulose membrane, blocked with 5% BSA or 5% non-fat milk and probed with indicated antibodies. Antibodies; anti-NDRG1 D6C2 mAb (Cell Signaling Technologies; #9408, 1:1000) and anti-rabbit IgG, HRP-linked antibody (Cell Signaling Technology; #7074). Monoclonal anti-β-actin-peroxidase (Sigma; A3854, 1:10000) was used to detect the loading control and ECL prime (Amersham; RPN2232) was used for signal detection.

### Immunoprecipitation

Immunoprecipitation of NDRG1 protein was performed using Dynabeads Co-Immunoprecipitation Kit (Invitrogen; 14321D). Following the kit specific protocol, rabbit α-NDRG1 antibody (Abcam; 196621) was conjugated to superparamagnetic Dynabeads M-270 Epoxy beads at 5μg per 1 mg of beads and incubated with protein extracted from freshly isolated tissue for 30 min at 4 °C. Purified NDRG1 was analyzed by Western blot, probed using a primary rabbit α-NDRG1 antibody (Abcam; 196621; 1:500) and secondary peroxidase conjugated α-rabbit IgG antibody (Jackson; 111-035-144, 1:500). Protein was detected with the chemiluminescent ECL Prime Western Blotting Detection Reagent (Cytiva; RPN2232).

### ELISA

For HEL-binding IgM^a^ ^90^ or IgG, plates were coated with 10 μg/ml HEL in carbonate buffer pH9.6 (20 mM Na_2_CO_3_, 35 mM NaHCO_3_), washed with wash buffer (PBS/Tween20), then blocked with PBS, 1%BSA. After washing, serially diluted serum samples were added in PBS/0.1%BSA, after incubation and washing, plates were incubated with 0.5 μg/mL biotinylated anti-IgM^a^ (BD Pharmigen, #553515; clone DS-1) or anti-IgG-HRP (Bethyl laboratories) in 1%FCS, 1% milk powder, 0.1% Tween20 and NaN_3_ in PBS, then washed. Avidin-alkaline phosphate (A7294) was added to the wells at 1:3000 in PBS/0.1%BSA and incubated, then washed, then plates developed with addition of 1 mg/ml Sigma 104 phosphatase substrate (#104105) in 50 mM Na_2_CO_3_, 0.5 mM MgCl_2_ pH9.8. Anti-IgG-HRP (Bethyl laboratories; A90–131P) was used to detect serum HEL-specific IgG. Absorbance was measured at 405 nm using a Bio-Rad model 550 Microplate reader. Background absorbance values were subtracted from absorbance readings before interpolation to standard curve using Hyperbola (X as concentration). Bethyl laboratories mouse IgG (E90–131), IgM (E90–101) and IgA (E90–103) quantification kits were used by protocol with serum titration of samples as follows: IgG 1:4000, IgM 1:2000, IgA 1:2000, developed using TMB substrate (Life Technologies; 00-4201-56) and detected at 450 nm. IL-2 concentration in the supernatant was quantified by Mouse Ready-Set-Go IL-2 ELISA kit (eBioscience; 88–7024) and developed using TMB substrate as above.

### Anti-nuclear antibody (ANA) staining

For ANA staining, serum samples were diluted at 1:100 in PBS and stored at −20 °C before incubation on Hep-2 cell coated slides (A.Menarini; 37806). Slides were washed with PBS, then water, then incubated with FITC-conjugated, goat anti-mouse IgG (ThermoFisher; 62–6511). After washing, slides were mounted and imaged on Nikon wide-field TE20000U Microscope (GFP channel: 20×, 400 ms) then analysed with automated cell segmentation and fluorescence intensity quantification with ImageJ. Positive scoring samples (2+) were manually cross-checked before scoring^92^^.^

### In vivo T cell anergy

CD45.2^+^ CD4^+^ T cells were isolated from WT OT-II+ or *Ndrg1*^*−/−*^ OT-II^+^ tg mice and adoptively transferred to CD45.1^+^ non-tg congenic recipients, with subsequent intravenous injection of 500μg endotoxin-free OVA peptide on day 1 (Insight Biotech; 21-51023-G (323–339)), as tested using Pierce LAL Chromogenic Endotoxin Quantitation kit (ThermoFisher; #88282). 5 days later, total splenic CD4^+^ cells were magnetically isolated from recipient mice and naïve unstimulated WT or *Ndrg1*^*−/−*^ OT-II^+^ control mice, before loading with CTV dye. As determined by flow cytometry, 4 × 10^4^ CD45.2^+^ Vα2^+^ OT-II^+^ CD4^+^ T cells were cultured in vitro with a titration of OVA peptide and 4 × 10^5^ irradiated splenocytes. IL-2 in culture supernatant at 48 h was measured by ELISA and proliferation of CD45.2^+^Vα2^+^ OT-II^+^ CD4^+^ T cells was detected by flow cytometry at 72 h.

### Statistics and reproducibility

GraphPad Prism Software was used for statistical analyses and unless otherwise specifically mentioned unpaired, two-tailed Student’s *t* tests were used for statistical comparison between groups, correcting for multiple comparisons using the Holm-Sidak method. Experimental groups were determined by genotype and were therefore not randomized and were not blinded. Experimental animals were not excluded from analysis except according to pre-specified experimental design on the basis of failed chimeric reconstitution or failed cell transfer. All experiments included age and sex matched controls, which were co-housed littermates wherever possible. Sample sizes were selected on the basis of previously published studies. Data shown is pooled from, or representative of replicate experiments as indicated in figure legends.

### Reporting summary

Further information on research design is available in the Nature Research Reporting Summary linked to this article.

## Supplementary information

Supplementary Information

Description of Additional Supplementary Files

Supplementary Data

Reporting Summary

## Data Availability

Raw RNA-Seq data are available in the Gene Expression Omnibus (https://www.ncbi.nlm.nih.gov/geo/) under accession code GSE135650. Supplementary Table 1 is provided in the Supplementary Data. Uncropped and unedited gel and blot images are available within Supplementary Fig. 4a–d. All other source data are available via the Oxford University Research Archive at https://ora.ox.ac.uk/objects/uuid:51f425ad-836f-4c3e-91d6-3321b0ab7b8b with the 10.5287/bodleian:gJnGGXymz.
