# Supplementary material for: NDRG1 is induced by antigen-receptor signaling but dispensable for B and T cell self-tolerance
Source: Commun Biol. 2022 Nov 10;5:1216. doi: 10.1038/s42003-022-04118-w (PMC9649591; doi:10.1038/s42003-022-04118-w)
Supplement: Supplementary file 1 — Supplementary Information [file 42003_2022_4118_MOESM1_ESM.pdf]

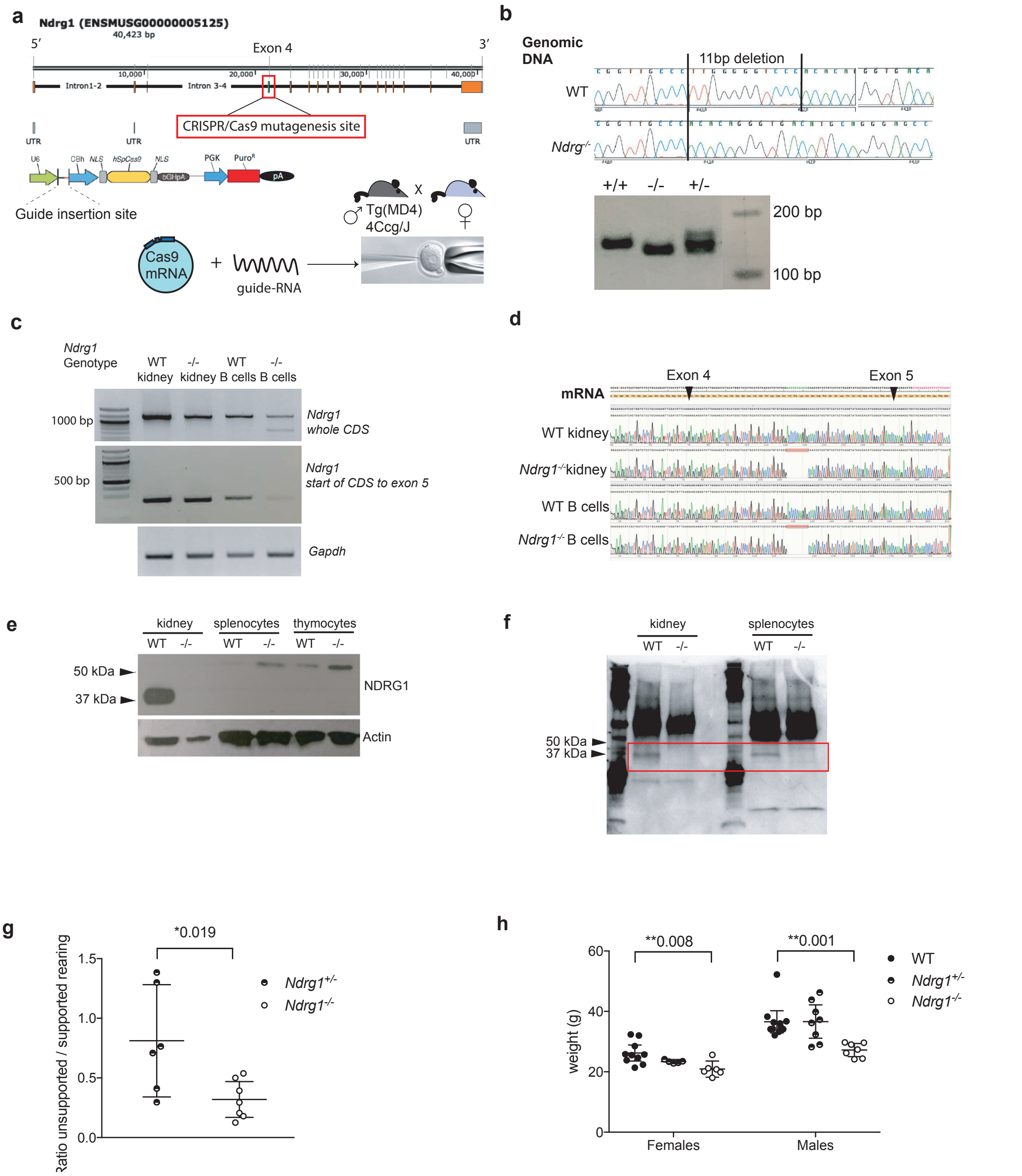

Supplemental Figure 1. Creation of a *Ndrg1*$^{-/-}$ mouse model by directed CRISPR-Cas9 mutagenesis.

a. Design of CRISPR/Cas9 guide RNAs targeting exon 4 (ENSMUSE00001241456) of the *Ndrg1* gene (ENSMUSG00000005125, upper panel). Complementary oligos of the guide sequences were cloned into pX330-Puro vector, which was used as template for guide-RNA generation. The guide-RNA was then microinjected with Cas9 mRNA into embryos derived from super-ovulated C57BL/6J females mated with Ig^{HEL} tg studs (lower panel). b. Sanger sequencing trace of genomic DNA at the mutagenesis site comparing WT and *Ndrg1*$^{-/-}$ mice (upper panel) using genomic primer set 1, and agarose gel electrophoresis of PCR-amplified genomic DNA at the CRISPR-mutagenesis site of *Ndrg1* gene (lower panel) using genomic primer set 2, cropped for presentation. c. Gel electrophoresis of PCR-amplified *Ndrg1* transcripts reverse transcribed from WT and *Ndrg1*$^{-/-}$ kidney or splenic B cell samples.

Top panel; bands amplified using mRNA set 1 primers spanning the entire *Ndrg1* coding sequence (CDS), the lower band in *Ndrg1*$^{-/-}$ B cells represents genomic DNA (*Wdr70*) contamination as confirmed by Sanger sequencing. Middle panel; bands amplified using mRNA set 2 primers spanning the start of the *Ndrg1* CDS to exon 5. Lower panel: Ga*pdh* expression. Gel images are cropped for presentation. d. Sanger sequencing of PCR products amplified in (c) (second panel) using the same primers. Sequences are aligned to *Ndrg1* mRNA sequence with exon starts labelled in orange. e. Western blot of protein lysates from WT and *Ndrg1*$^{-/-}$ samples using N-terminal binding antibody; top blot shows kidney samples from 3 mice per group. NDRG1 detected at 37kDa, Bottom blot shows kidney, splenocyte and thymocyte lysates, highlighting that NDRG1 protein amounts are below the threshold of detection in splenocyte and thymocyte samples, Bands > 50kDa are attributed to non-specific antibody binding. Actin was used as loading control for both blots (lower panels). Gel images are cropped for presentation. f. Immunoprecipitation of NDRG1 proteins and blotting of WT and *Ndrg1*$^{-/-}$ kidney and splenic protein lysates, bands > 50kDa and < 20kDa are attributed to non-specific antibody binding. g. Ratio of unsupported to supported rearing in *Ndrg1*$^{+/-}$ and *Ndrg1*$^{-/-}$ adult mice, circles are individual mice, line represents mean and 95% CI. h. Weights of WT, *Ndrg1*$^{+/-}$ and *Ndrg1*$^{-/-}$ adult mice aged 14 - 25 weeks of age, where circles are individual mice, lines represent mean and 95% CI.

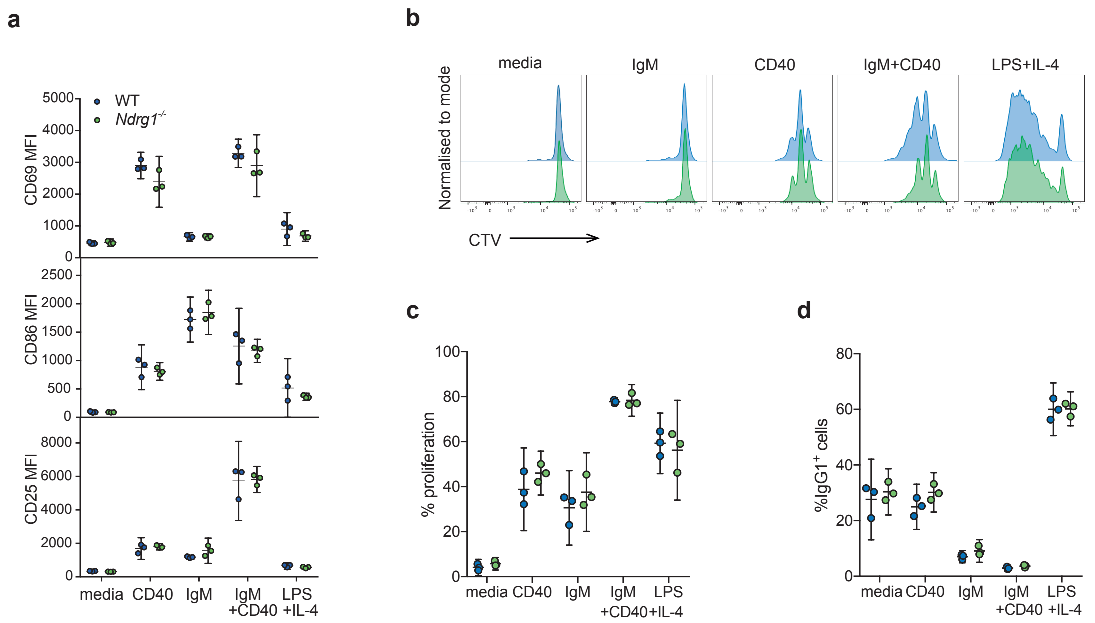

Supplemental Figure 2. *Ndrg1*$^{-/-}$ mice respond normally to foreign antigen.
a. Median fluorescence intensities (MFIs) corresponding to activation marker expression on WT and *Ndrg1*$^{-/-}$ primary B220^{+} splenic B cells stimulated with media alone, or combinations of anti-CD40 2.5μg/ml, anti-IgM 2.5μg/ml, LPS 10μg/ml and IL-4 10ng/ml for 24 hours at 37°C *ex vivo*. b. Flow cytometric plots of CTV dilution in B220^{+} splenocytes stimulated with the same conditions as (a) for 72h, with quantification of proliferation (c) and class-switching (d). Results are representative of two independent experiments: circles are representative of individual mice and lines are means with 95% CI error bars.

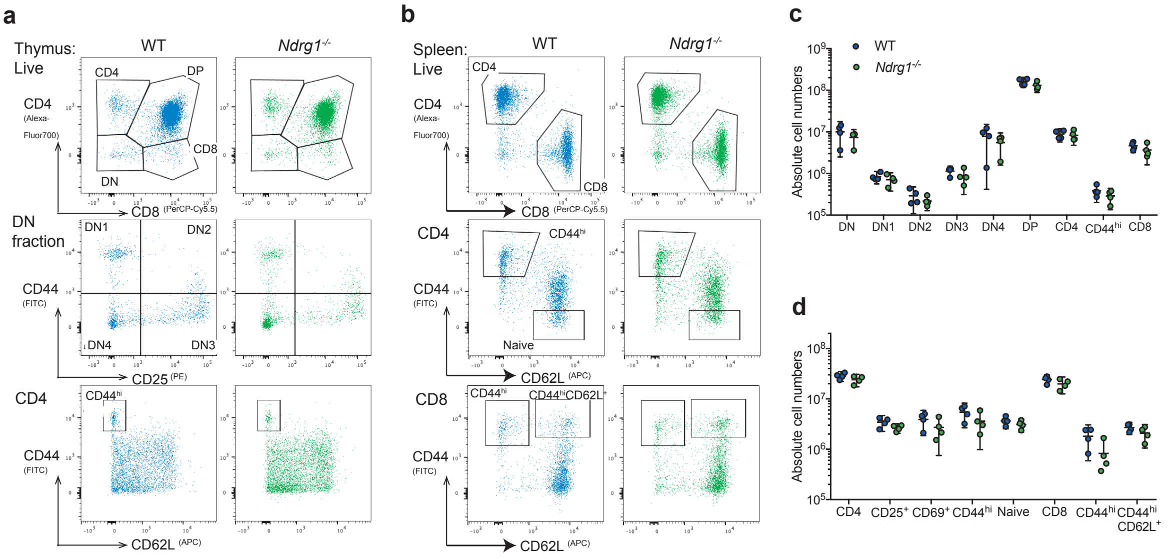

Supplemental Figure 3. T cell development is normal in *Ndrg1*$^{-/-}$ mice

a. Representative flow cytometry plots of WT and *Ndrg1*$^{-/-}$ T cell development in the thymus, and b. peripheral T cell populations in the spleen. c. Quantification of WT and *Ndrg1*$^{-/-}$ thymic T cell subsets as gated in (a). d. Numbers of WT and *Ndrg1*$^{-/-}$ splenic T cell populations as gated in (b). Results are representative of three independent experiments, each with 3-6 mice per group individually represented by each data point, where lines show means and bars 95% CI.

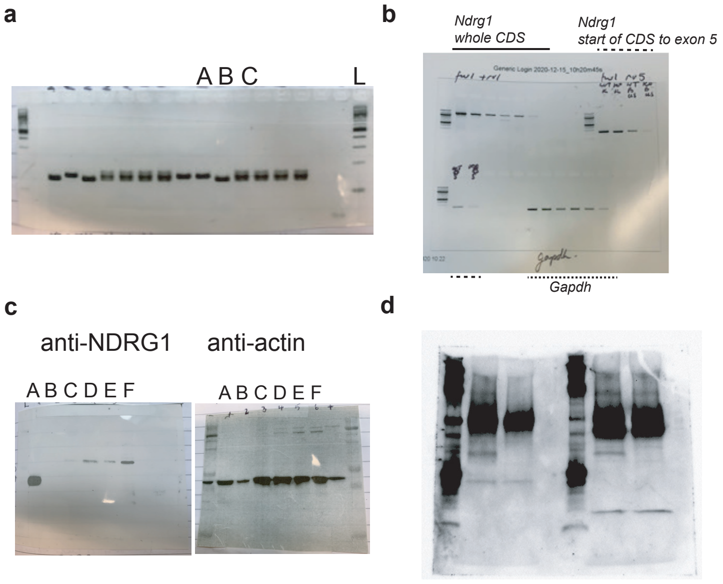

Supplemental Figure 4. Unedited and uncropped gels and blots

a. Uncropped DNA gel from (Supplemental Fig. 1b). Lanes marked with A, B, C and L represent used data shown in (Supplemental Fig. 1b). b. Uncropped cDNA gel from (Supplemental Fig. 1c), with targets identified. In each case, the first four lanes of each target were cropped for use in (Supplemental Fig. 1c). c. Uncropped and unedited immunoblots targeting NDRG1 and actin as indicated from (Supplemental Fig. 1e). Lanes marked with A-F mark those used in (Supplemental Fig. 1e). d. Unedited immunoblot from immunoprecipitation in (Supplemental Fig. 1f).
