# Supplementary material for: NDRG1 is induced by antigen-receptor signaling but dispensable for B and T cell self-tolerance
Source: Commun Biol. 2022 Nov 10;5:1216. doi: 10.1038/s42003-022-04118-w (PMC9649591; doi:10.1038/s42003-022-04118-w)
Supplement: Supplementary file 2 — Description of Additional Supplementary Files [file 42003_2022_4118_MOESM2_ESM.pdf]

Description of Additional Supplementary Files

File name: Supplementary Data

Description: Top differentially expressed genes between anergic and naïve follicular B cells. Genes differentially expressed over 1 log2FoldChange and below a significance threshold of adjusted p value 0.05 between anergic and naïve follicular B cells as determined by DESeq2. Genes are sorted here by log2FoldChange.
