# Supplementary material for: NDRG1 is induced by antigen-receptor signaling but dispensable for B and T cell self-tolerance
Source: Commun Biol. 2022 Nov 10;5:1216. doi: 10.1038/s42003-022-04118-w (PMC9649591; doi:10.1038/s42003-022-04118-w)
Supplement: Supplementary file 4 — Reporting Summary [file 42003_2022_4118_MOESM4_ESM.pdf]

nature research

Corresponding author(s): COMMSBIO-21-3316A Katherine Bull

Last updated by author(s): Oct 2, 2022

Reporting Summary

Nature Research wishes to improve the reproducibility of the work that we publish. This form provides structure for consistency and transparency in reporting. For further information on Nature Research policies, see our Editorial Policies and the Editorial Policy Checklist.

Statistics

For all statistical analyses, confirm that the following items are present in the figure legend, table legend, main text, or Methods section.

n/a	Confirmed	
☐	☒	The exact sample size (*n*) for each experimental group/condition, given as a discrete number and unit of measurement
☐	☒	A statement on whether measurements were taken from distinct samples or whether the same sample was measured repeatedly
☐	☒	The statistical test(s) used AND whether they are one- or two-sided<br>*Only common tests should be described solely by name; describe more complex techniques in the Methods section.*
☐	☒	A description of all covariates tested
☐	☒	A description of any assumptions or corrections, such as tests of normality and adjustment for multiple comparisons
☐	☒	A full description of the statistical parameters including central tendency (e.g. means) or other basic estimates (e.g. regression coefficient) AND variation (e.g. standard deviation) or associated estimates of uncertainty (e.g. confidence intervals)
☐	☒	For null hypothesis testing, the test statistic (e.g. F, t, r) with confidence intervals, effect sizes, degrees of freedom and P value noted<br>*Give P values as exact values whenever suitable.*
☒	☐	For Bayesian analysis, information on the choice of priors and Markov chain Monte Carlo settings
☒	☐	For hierarchical and complex designs, identification of the appropriate level for tests and full reporting of outcomes
☒	☐	Estimates of effect sizes (e.g. Cohen's *d*, Pearson's *r*), indicating how they were calculated

Our web collection on statistics for biologists contains articles on many of the points above.

Software and code

Policy information about availability of computer code

Data collection	RNA-Seq data from Fig 1 are publicably available. A count matrix was used as input for the below analyses.
Data analysis	DESeq2 v.1.28.1 was used to perform differential expression analysis for RNA-Seq from publicably available data in R. EnhancedVolcano (1.6.0) was used for visualisation.

For manuscripts utilizing custom algorithms or software that are central to the research but not yet described in published literature, software must be made available to editors and reviewers. We strongly encourage code deposition in a community repository (e.g. GitHub). See the Nature Research guidelines for submitting code & software for further information.

Data

Policy information about availability of data

All manuscripts must include a data availability statement. This statement should provide the following information, where applicable:

- Accession codes, unique identifiers, or web links for publicly available datasets
- A list of figures that have associated raw data
- A description of any restrictions on data availability

Raw RNA-Seq data is available in the Gene Expression Omnibus (https://www.ncbi.nlm.nih.gov/geo/) under accession code GSE135650. Source data for Figures 2-7 and Supplemental figures 1-4 will be made available via the Oxford University Research Archive (https://ora.ox.ac.uk).

Field-specific reporting

Please select the one below that is the best fit for your research. If you are not sure, read the appropriate sections before making your selection.

☒ Life sciences ☐ Behavioural & social sciences ☐ Ecological, evolutionary & environmental sciences

For a reference copy of the document with all sections, see nature.com/documents/nr-reporting-summary-flat.pdf

Life sciences study design

All studies must disclose on these points even when the disclosure is negative.

Sample size	Sample sizes were selected on the basis of previously published studies
Data exclusions	Experimental animals were not excluded from analysis except according to pre-specified experimental design on the basis of failed chimeric reconstitution or failed cell transfer.
Replication	The number of repeats for each figure is detailed within the legend. All attempts at replication were successful.
Randomization	Experimental groups were determined by genotype and were therefore not randomized and were not blinded. All experiments included age and sex matched controls, which were co-housed littermates wherever possible.
Blinding	Experimental groups were determined by genotype and were therefore not randomized and were not blinded.

Reporting for specific materials, systems and methods

We require information from authors about some types of materials, experimental systems and methods used in many studies. Here, indicate whether each material, system or method listed is relevant to your study. If you are not sure if a list item applies to your research, read the appropriate section before selecting a response.

Materials & experimental systems

n/a	Involved in the study	
☐	☒	Antibodies
☐	☒	Eukaryotic cell lines
☒	☐	Palaeontology and archaeology
☐	☒	Animals and other organisms
☒	☐	Human research participants
☒	☐	Clinical data
☒	☐	Dual use research of concern

Methods

n/a	Involved in the study	
☒	☐	ChIP-seq
☐	☒	Flow cytometry
☒	☐	MRI-based neuroimaging

Antibodies

Antibodies used	The following antibodies used during flow cytometric staining were from Biolegend; anti-B220 (103236, 103232, 103212, 103243), anti-CD19 (115528, 115530), Zombie Aqua live/dead (423102, 423106), anti-IgM (406508, 406512), anti-IgD (405704, 405716, 405708), anti-IgDa (406104), anti-IgMa (408608), anti-CD23 (101614), anti-CD93 (136510), anti-CD21 (123418, 123412), anti-CD24 (101820, 101836), anti-CD93 (136510), anti-CD5 (100629), anti-CD86 (105028), anti-CD44 (103020, 103006), anti-CD25 (102008), anti-CD4 (100430), anti-CD8a (100734), anti-CD62L (104412), anti-CD3 (100214, 100330, 100328), anti-CD69 (104530), anti-CD45.1 (110730, 110722, 110708), anti-CD45.2 (109841, 109818, 109824), anti-BAFF-R (134103), anti-CD95 (152604) and anti-TCRV[illegible]2 (127806). The following antibodies were from BD Pharmingen; anti-CD21 (563176), anti-BP-1 (553735), anti-IgMa (553516), anti-CD43 (562865), anti-IgM (553437), phospho-PLC[illegible]2 (558498), phospho-BLNK (558443), anti-BrdU (364108), anti-CXCR5 (145504), anti-Bcl2 (633506), anti-Bcl2 quantification kit (556537), anti-CD95 (557653) and anti-IgG1 (563285). The following antibodies were from eBioscience anti-IgM (48-5890-82), anti-CD4 (25-0041-82), phospho-ERK (53-9109-42) and phospho-SYK (12-9014-42). CellTraceViolet cell proliferation kit (C34557) was from ThermoFisher. Anti-Bim was from CST NEB (10408S).<br><br>For ELISAs, Bethyl laboratories mouse IgG (E90-131), IgM (E90-101) and IgA (E90-103) quantification kits & Mouse Ready-Set-Go IL-2 ELISA kit (eBioscience; 88-7024) were used. For immunoblotting, rabbit α-NDRG1 antibody (Abcam; 196621), anti-NDRG1 D6C2 mAb (Cell Signaling Technologies; #9408, 1:1000) were used.
Validation	All antibodies used were validated by the relevant manufacturer on mouse target cells of interest where available, or mouse cell lines/tissues/serum where not, each for the relevant application (flow cytometry, immunoblot or ELISA). These antibodies were generally also chosen based on previous citations. Most of these antibodies have been validated within the lab for use with mouse cells and tissues and thus cited in previous lab publications.

Eukaryotic cell lines

Policy information about cell lines

Cell line source(s)	A20 B cell line
Authentication	the A20 B cell line was not authenticated
Mycoplasma contamination	the A20 B cell line was not tested for mycoplasma contamination
Commonly misidentified lines (See ICLAC register)	The A20 cell line is not a commonly misidentified line

Animals and other organisms

Policy information about studies involving animals; ARRIVE guidelines recommended for reporting animal research

Laboratory animals	Mice: C57BL/6JOlaHsd,. IgHEL (C57BL/6-Tg(MD4)4Ccg/J), soluble HEL (sHEL) tg (C57BL/6-Tg(ML5)5Ccg/J) and OT-II tg mice (C57BL/6-Tg(TcraTcrb) 425Cbn/Crl), Ndrg1-/- mice were generated by CRISPR/Cas9 directed mutagenesis on a C57BL/6J background as described in this manuscript. All experiments included age and sex matched controls, which were co-housed littermates wherever possible. Sample sizes were selected on the basis of previously published studies.
Wild animals	The study did not involve wild animals
Field-collected samples	The study did not involve field collected samples
Ethics oversight	All procedures involving animals were performed in accordance with the Animals (Scientific Procedures) Act 1986, amended 2012, with procedures reviewed by the clinical medicine Animal Care and Ethical Review Body and conducted under Home Office Project License, P79A4C5BA.

Note that full information on the approval of the study protocol must also be provided in the manuscript.

Flow Cytometry

Plots

Confirm that:

☒ The axis labels state the marker and fluorochrome used (e.g. CD4-FITC).

☒ The axis scales are clearly visible. Include numbers along axes only for bottom left plot of group (a 'group' is an analysis of identical markers).

☒ All plots are contour plots with outliers or pseudocolor plots.

☒ A numerical value for number of cells or percentage (with statistics) is provided.

Methodology

Sample preparation	Mouse organs were processed to single cell suspensions by either processing through a 70um cell strainer (Spleen, LN) or by syringe and needle for bone marrow. Suspensions were then either directly processed for flow cytometry in buffer containing Hepes, sodium azide and 2% FCS or treated with red blood cell lysis for removal of erythrocytes.
Instrument	BD FACS CANTO10c
Software	BD DIVA and FlowJo
Cell population abundance	Between 1-2 x 10^6 cells are stained per sample. At least 50,000 cells are acquired per sample. WBC counts, determined using haemocytometer, are used to back-calculate absolute numbers from flow cytometry cell percentages. For expression analyses there were at least 100 cells within the gate of interest.
Gating strategy	All gating strategies are detailed either within the figures or legends within the manuscript. All flow cytometry data shown follows the initial gating scheme of singlets (FSC-H, FSC-A), then on SSC-A, FSC-A for either white blood cells for number analyses or a tighter lymphocyte only gate for population level analyses. A viability gate then generally follows this gate and the following gates are all described specifically for each figure within the manuscript. See also source data.

☒ Tick this box to confirm that a figure exemplifying the gating strategy is provided in the Supplementary Information.
